# Therapeutic Drug Monitoring of Tyrosine Kinase Inhibitors Using Dried Blood Microsamples

**DOI:** 10.3389/fonc.2022.821807

**Published:** 2022-03-22

**Authors:** Nick Verougstraete, Veronique Stove, Alain G. Verstraete, Christophe P. Stove

**Affiliations:** ^1^ Laboratory of Toxicology, Department of Bioanalysis, Faculty of Pharmaceutical Sciences, Ghent University, Ghent, Belgium; ^2^ Department of Laboratory Medicine, Ghent University Hospital, Ghent, Belgium; ^3^ Department of Diagnostic Sciences, Faculty of Medicine and Health Sciences, Ghent University, Ghent, Belgium

**Keywords:** tyrosine kinase inhibitors, therapeutic drug monitoring, microsampling, dried blood microsamples, oncology drugs

## Abstract

Therapeutic drug monitoring (TDM) of tyrosine kinase inhibitors (TKIs) is not yet performed routinely in the standard care of oncology patients, although it offers a high potential to improve treatment outcome and minimize toxicity. TKIs are perfect candidates for TDM as they show a relatively small therapeutic window, a wide inter-patient variability in pharmacokinetics and a correlation between drug concentration and effect. Moreover, most of the available TKIs are susceptible to various drug-drug interactions and medication adherence can be checked by performing TDM. Plasma, obtained *via* traditional venous blood sampling, is the standard matrix for TDM of TKIs. However, the use of plasma poses some challenges related to sampling and stability. The use of dried blood microsamples can overcome these limitations. Collection of samples *via* finger-prick is minimally invasive and considered convenient and simple, enabling sampling by the patients themselves in their home-setting. The collection of small sample volumes is especially relevant for use in pediatric populations or in pharmacokinetic studies. Additionally, working with dried matrices improves compound stability, resulting in convenient and cost-effective transport and storage of the samples. In this review we focus on the different dried blood microsample-based methods that were used for the quantification of TKIs. Despite the many advantages associated with dried blood microsampling, quantitative analyses are also associated with some specific difficulties. Different methodological aspects of microsampling-based methods are discussed and applied to TDM of TKIs. We focus on sample preparation, analytics, internal standards, dilution of samples, external quality controls, dried blood spot specific validation parameters, stability and blood-to-plasma conversion methods. The various impacts of deviating hematocrit values on quantitative results are discussed in a separate section as this is a key issue and undoubtedly the most widely discussed issue in the analysis of dried blood microsamples. Lastly, the applicability and feasibility of performing TDM using microsamples in a real-life home-sampling context is discussed.

## Introduction: TDM of TKIs

Therapeutic drug monitoring (TDM) refers to the determination of therapeutic drug concentrations in biological fluids, mostly serum, plasma or blood, with the aim of improving individual treatment by dose adjustment. In clinical practice, TDM is already routinely performed for several classes of therapeutic compounds, such as immunosuppressants, antibiotics and anti-epileptic drugs. Although evidence is accumulating that there also lies significant potential in the field of cancer treatment, TDM is not yet routinely performed for oncology drugs (1, 2).

Constitutive over-activation of tyrosine kinases (e.g. *via* mutations or *via* constitutive ligand-mediated activation) is known to underlie uncontrolled cell growth and proliferation, resistance to apoptosis and lack of differentiation. Tyrosine kinase inhibitors (TKIs) are a relatively new class of targeted cancer therapy specifically inhibiting tyrosine kinases responsible for deregulation of intracellular signaling pathways in tumor cells (3). A wide array of TKIs has become available that is increasingly being used for the treatment of various malignant diseases, including chronic myeloid leukemia (CML), gastrointestinal stromal tumor (GIST), non-small-cell lung carcinoma (NSCLC), renal cell carcinoma (RCC) and melanoma. In contrast to most classical cytotoxic agents, TKIs are administered orally enabling outpatient treatment. Besides, TKIs are usually given in non-individualized fixed doses (4).

TKIs have a relatively small therapeutic window and show high inter-individual pharmacokinetic variability after oral administration, caused by variation in absorption, distribution, metabolism and excretion (2, 4, 5). Following a given dose, a wide range of plasma concentrations among different patients are to be expected, which can possibly affect therapeutic responses and adverse effects (6). Bioavailability of TKIs depends on drug formulation, gastrointestinal absorption, concomitant food intake (e.g. increased bioavailability of nilotinib and pazopanib when administered under fed conditions (7)) and first-pass metabolism by the liver (8). As the majority of TKIs are primarily metabolized *via* the cytochrome P450 3A4 isoenzyme, this makes them prone to drug-drug interactions. The latter is highly relevant for TKIs as polypharmacy is common in cancer patients: for example, in acute myeloid leukemia (AML) treatment clinically relevant pharmacokinetic interactions have been described between the FLT3 inhibitors (midostaurin and gilteritinib) and azole antifungals (9, 10). Genetic polymorphisms, age, lifestyle factors (e.g. influence of smoking on erlotinib metabolic clearance (11)) and comorbidities also have an influence on the CYP enzymes’ activity (2, 4, 12). Moreover, some of the TKIs are substrates for drug transporters, e.g. erlotinib is a P-glycoprotein substrate (12).

In the context of chronic conditions with a need for long-term oral treatment, medication adherence is very important and a major determinant in the therapeutic success of TKIs. Again, this can be assessed by monitoring trough TKI levels (6, 13, 14). Patients’ adherence to oral anticancer drugs is highly variable, with reported rates varying from 100 down to 20% (15). In CML, poor adherence is the main reason for the lack of obtaining a molecular response (16). For most of the available TKIs, studies have already defined exposure-response relationships. On the one hand, underexposure may result in reduced efficacy with therapy failure and/or more rapid development of TKI resistance. On the other hand, if the exposure is too high, the risk of treatment-related toxicity, with occurrence of adverse effects is increased (6). This, in turn, may negatively impact adherence. Given the (once or twice) daily chronic administration of most TKIs, single steady-state trough levels (i.e. just before the next dose) allow estimating TKI exposure (2). Steady-state levels are achieved after 4-5 times the elimination half-life of the drug (4). For newer TKIs, exposure-response relationships and suitable pharmacokinetic thresholds associated with favorable outcome still need to be defined (5, 17–19). Furthermore, feasible dose-adaptation strategies should exist (4).

For the above-mentioned reasons, TKIs have been suggested as perfect candidates for routinely performing TDM. Based on evidence level, TDM is currently recommended for axitinib, gefitinib, imatinib, pazopanib, sunitinib and trametinib (5). Recently, the International Association of Therapeutic Drug Monitoring and Clinical Toxicology (IATDMCT) has published a consensus guideline for TDM of imatinib (20). The evidence level for TDM for the TKIs alectinib, crizotinib, erlotinib, gilteritinib, nilotinib and vemurafenib is categorized as “potentially useful” because exposure-response relationships and target concentrations have been established but TDM feasibility studies have not yet been performed. For a wide range of additional TKIs, including among others bosutinib, dasatinib, ibrutinib, midostaurin and ponatinib evidence is still in the exploratory phase. TDM of cobimetinib, entrectinib, lapatinib and larotrectinib is not recommended (5).

Lastly, considering the high costs of these drugs and the high consequences of subtherapeutic concentrations, efforts to ensure their therapeutic potential should be made. Related to this, a shift from a TKI fixed dosing paradigm to individual dosing based on TDM results is recommended. In the context of personalized cancer treatment, TDM is a relatively new strategy that could be valuable to optimize TKI dosage, resulting in better patient outcome.

TKI therapeutic ranges are typically expressed as plasma concentrations, hence plasma, obtained *via* conventional venous blood sampling, is the standard matrix for TDM of TKIs. However, the use of plasma as a matrix poses some challenges related to sampling and stability. Venous blood is acquired *via* an invasive venous blood sampling procedure, performed by qualified medical personnel. For cancer patients, this conventional venous sampling may be challenging because of difficulties to access peripheral blood vessels due to the frequent blood sample collection, combined with the chemotherapy administration. Moreover, the preparation of plasma requires a centrifugation step of whole blood and for most TKIs refrigerated storage and transport of the sample (blood or plasma) is needed (21). The latter is relevant as TDM for TKIs is typically only performed at specialized centers, requiring transportation from the sampling site to a dedicated clinical lab. Stability can also be a key factor. Instability is for instance relevant for ibrutinib, which is subject to rapid degradation in plasma at room temperature and at 4°C (22, 23). Moreover, diminished stability in plasma samples stored at room temperature has also been reported for lapatinib and axitinib (<24 hours) (24) and sunitinib (<48 hours) (25).

The above-mentioned issues can be overcome using alternative sampling strategies, in which traditional matrices are collected *via* an alternative approach. Dried blood microsampling appears to be the most promising option for application of TDM of TKIs (1). It refers to the collection of a small amount of blood (typically <50 µL), most often following a finger-prick using an automatic (single-use) lancet. Dried blood microsamples are generally dried for 2-3 hours at ambient temperature. Given the minimal invasiveness and the low volume of sample collected, dried blood microsampling is considered more patient-friendly than conventional venous blood sampling. This is particularly relevant for use in pediatric populations. Moreover, given the convenient and simple sampling method, (micro)samples can be collected by the patients themselves outside the healthcare environment. This is advantageous for outpatients as they are not required to go to a medical center for a blood draw. Furthermore, sampling at predefined times (e.g. trough levels for most TDM applications) is also more convenient in a home-setting and will not lead to a delay in drug intake. Hence, it can be concluded that, overall, microsampling complies with a patient-centric view when it relates to sample collection. In addition, microsampling is also a valuable tool to investigate the pharmacokinetics of TKIs (12, 26), especially for the pharmacokinetic assessment of new investigational TKIs during clinical trials (27, 28). **Table 1** provides a summary of the advantages and challenges associated with dried blood microsampling for TDM of TKIs.

**Table 1:** Advantages and challenges associated with TKI (dried) microsampling.

Advantages	Challenges
Minimally invasive sampling	Correlation with (venous) plasma concentrations
Small sample volume	Hematocrit effects: differential spreading of blood samples (DBS), impact on recovery and blood-to-plasma ratio
Convenient and cost-effective transport and storage	Influence of spotted volume & spot homogeneity (DBS)
No need for centrifugation step	Sensitive analytical techniques required
Compound stability	Dilution of samples >ULOQ
Home sampling	Lack of proficiency testing programs
Less biohazardous	Risk of inadequate sampling
	Extra validation steps needed
	Sampling material is more expensive than blood tubes
	Not compatible with track systems in clinical labs
	Need for drying for 2 hours

DBS, dried blood spots; ULOQ, upper limit of quantification.

The most widespread and well-known microsampling technique is dried blood spot (DBS) sampling. DBS are generated by collecting a drop of capillary blood, derived from a finger- or heel-prick, on special filter paper. In a typical DBS workflow, a 3-6 mm diameter disc is punched from the DBS, followed by extraction of this subpunch. While there is a plethora of papers concerning TDM *via* DBS (29–33), relatively few of these deal with oral anticancer drugs or TKIs (34), although recently there is an increased interest. Apart from conventional DBS, other microsampling devices are also being explored for TKI monitoring. E.g., we recently reported on the determination of a TKI panel in samples collected *via* volumetric absorptive microsampling (VAMS) (35). Briefly, VAMS is a sampling technique in which a fixed volume of blood is wicked up by an absorbent tip attached to a plastic handler, used as an alternative to classical DBS sampling. **Table 2** gives an overview of published methods to determine TKIs in different dried blood microsamples. In what follows we will discuss these methods, along with the benefits and challenges, as outlined in **Table 1**.

**Table 2:** Overview of published microsample TKI-based methods in alphabetical order of the first author.

Reference	Micro-sampling technique	Included TKIs	Assay range	Complete DBS vs DBS punch	Sample preparation	Internal standard	Application	Mean Hct (+ range) of study population
Antunes et al. (36)	DBS	Imatinib	50-4000 ng/mL	Punch: 6 mm d	Direct extraction with MeOH	SIL	Comparison between DBS and plasma samples from 50 CML patients	0.36 (0.29-0.43)
Boons et al. (37)	DBS	Nilotinib	17-4100 ng/mL	Punch: 8 mm d	Direct extraction with MeOH	SIL	Comparison between 40 DBS and plasma samples from 20 CML patients	0.41
de Wit et al. (38)	DBS	Pazopanib	0.1-50 µg/mL	Punch: 4 mm d	Direct extraction with FA and MeOH	SIL	Comparison between DBS and plasma samples from 12 mRCC patients	0.45 (0.40-0.49)
Iacuzzi et al. (39)	DBS	Imatinib	50-7500 ng/mL	Punch: 3 mm d	Direct extraction with 0.1% FA in MeOH	SIL	Comparison between 55 DBS (both from venous blood and from finger prick) and plasma samples from 26 GIST patients	0.38 (0.26-0.44)
		Norimatinib	10-1500 ng/mL					
Irie et al. (40)	DBS	Gefitinib	37.5-2400 ng/mL	Punch: 3 mm d	Direct extraction with MeOH	Erlotinib	Comparison between DBS and plasma samples from 10 NSCLC patients	0.39 (0.32-0.42)
Kralj et al. (41)	DBS	Imatinib	50-5000 ng/mL	Complete: 10 µL	Direct extraction with 0.1% FA in MeOH	SIL	Comparison between 24 DBS and plasma samples from CML patients	*
		Nilotinib	50-5000 ng/mL					
		Dasatinib	2.5-250 ng/mL					
Lee et al. (42)	DBS	Radotinib	5-2000 ng/mL	Punch: 6 mm d	Direct extraction with “extracting solvent”	*	Comparison between DBS and plasma samples from 45 CML patients	0.41
Martin et al. (28)	DBS	R406 (active metabolite of fostamatinib)	2.5-2500 ng/mL	*	*	SIL	Comparison between plasma, venous whole blood, finger prick-drawn DBS and pipette-drawn DBS samples from 24 patients in a phase I clinical dose study	*
Mukai et al. (43)	DPS	Dasatinib	1-200 ng/mL	Complete: 40 µL	Extraction with MeOH/ACN (50/50, v/v) followed by SPE and evaporation to dryness	SIL	Comparison between 96 (venous) DPS and plasma samples	NA
		Ponatinib	1-200 ng/mL					
		Ibrutinib	2-400 ng/mL					
		Bosutinib	5-1000 ng/mL					
		Imatinib	20-4000 ng/mL					
		Nilotinib	20-4000 ng/mL					
Nijenhuis et al. (26, 44)	DBS	Vemurafenib	1-100 µg/mL	Punch: 3 mm d	Direct extraction with MeOH/ACN (50/50, v/v)	SIL	Comparison between 43 DBS and plasma samples from 8 melanoma patients (26)	0.40 (0.27-0.49) (26)
Parra-Guillen et al. (12)	DBS	Erlotinib	10-10000 ng/mL	Punch: 3 mm d	Direct extraction with MeOH/water (50/50, v/v)	SIL	Comparison between DBS and plasma samples from 36 patients with advanced NSCLC	*
		Metabolite OSI-420	2.5-2500 ng/mL					
Verheijen et al. (45)	DBS	Pazopanib	1-50 µg/mL	Punch: 3 mm d	Direct extraction with FA and subsequent MeOH	SIL	Comparison between 329 DBS and plasma samples from 30 patients with advanced solid tumors	0.40 (0.36-0.48)
Verougstraete and Stove (35)	VAMS	Bosutinib	5-675 ng/mL	NA	Pre-wetting VAMS tips with water followed by sonication, LLE with methyl-t-butyl ether and evaporation to dryness	SIL	Comparison between 27 (venous) DBS and liquid whole blood samples from oncology patients	0.39 (0.20-0.49)
		Dasatinib	0.5-450 ng/mL					
		Gilteritinib	25-675 ng/mL					
		Ibrutinib	5-675 ng/mL					
		Imatinib	10-2250 ng/mL					
		Midostaurin	30-2250 ng/mL					
		Nilotinib	10-2250 ng/mL					
		Ponatinib	1-450 ng/mL					
Xu et al. (27)	DBS	MK-1775 (adavosertib)	2-1000 ng/mL	Punch: 3 mm d	Direct extraction with 85% ACN – 10mM ammonium formate	SIL	Comparison between DBS and plasma samples from 12 solid tumor patients	(0.32-0.48)
Zimmermann et al. (46)	VAMS	Afatinib	2-500 ng/mL	NA	Rehydration of VAMS tips with water followed by addition of ACN as extraction solution and evaporation of the supernatant	SIL	24 VAMS samples from 5 patients with various malignancies	0.38 (0.31-0.41)
		Axitinib	2-500 ng/mL					
		Bosutinib	2-500 ng/mL					
		Cabozatinib	6-1500 ng/mL					
		Dabrafenib	6-1500 ng/mL					
		Lenvatinib	2-500 ng/mL					
		Nilotinib	6-1500 ng/mL					
		Osimeritinib	6-1500 ng/mL					
		Ruxolitinib	2-500 ng/mL					
		Trametinib	2-500 ng/mL					

ACN, acetonitrile; CML, chronic myeloid leukemia; d, diameter; DBS, dried blood spot; DPS, dried plasma spot; FA, formic acid; GIST, gastro-intestinal stromal tumor; LLE, liquid-liquid extraction; MeOH, methanol; NA, not applicable; mRCC, metastatic renal cell carcinoma; NA, not applicable; NSCLC, non-small cell lung cancer; SIL, stable isotopically labeled; TKI, tyrosine kinase inhibitor; VAMS, volumetric absorptive microsampling.

*information not described in the paper.

## Methodological Aspects of Dried Blood Microsample Methods for TKI TDM

Despite the many advantages associated with dried blood microsampling, quantitative analyses on these matrices requires tackling a few hurdles, which will be discussed in this section. The hematocrit (Hct)-effect will be discussed in a separate paragraph as this is a key issue and undoubtedly the most widely discussed issue in the analysis of dried blood microsamples (47–49). To guide successful incorporation of TDM methods in routine, the IATDMCT published an extensive guideline covering the development and validation of DBS-based methods, including both classical validation parameters and dried blood specific parameters. Furthermore, this guideline provides guidance on how to deal with specific dried blood associated issues and pitfalls during method development (50). In essence, it shouldn’t matter whether a TKI is determined in liquid or dried blood, implying that concentrations measured in a dried blood microsample should mimic those obtained from liquid blood. If not, this points at a methodological issue associated with the dried blood-based method, which should be addressed.

### a. Sample Preparation

For plasma-based methods for TKI TDM, simple protein precipitation with organic solvents is the most commonly used sample pretreatment approach (51). In dried blood microsample bioanalysis, sample preparation plays a pivotal role, as extractability issues (e.g. differences in recovery between aged *versus* fresh samples, or between samples with a different Hct) may be detrimental for a method’s usefulness in clinical practice. Several sample preparation approaches have been described for TKI determination in dried blood microsamples. The easiest extraction method includes a single-step extraction by addition of an organic solvent or a mixture of solvents to the microsample. After subsequent shaking and centrifugation, the supernatant can be injected on the analytical column (12, 27, 36–42, 44, 45). This is the most preferred approach as the sample handling is simple and fast. However, the high amount of organic solvent in these extracts may influence the chromatographic run. If sensitivity allows, the extracts can be diluted with water prior to injection to eliminate this solvent-effect on chromatography (38, 44, 45). The concurrent determination of several TKIs may require a more sophisticated sample preparation strategy. For example, the sample preparation of dried plasma spots (DPS) for the measurement of bosutinib, dasatinib, ibrutinib, imatinib, nilotinib and ponatinib consisted of a liquid extraction step followed by solid-phase extraction and subsequent drying (43). In two VAMS-based methods, VAMS samples were pre-wetted with an aqueous solution prior to the addition of an organic solvent for extraction (35, 46). Overall, a robust extraction procedure is key to the reliability of a method using dried blood microsamples.

### b. Analytics

All reported microsample-based TKI methods used in-house developed liquid chromatography coupled to tandem mass spectrometry (LC-MS/MS) as analytical technique. Concentration ranges in dried blood microsamples are similar to the ones observed in plasma matrices. A high sensitivity is required to attain adequate lower limits of quantitation (LLOQs) – ideally not higher than 50% of the lower end of the therapeutic range. Although current new-generation MS detectors offer a high sensitivity, this may still be a challenge when aiming at detecting TKIs with trough levels in the low-ng/mL range in dried blood microsamples corresponding to merely 3 to 10 µL blood (49, 51). Examples include dasatinib and talazoparib, with median reported trough levels of 2.61 ng/mL and 3.54 ng/mL, respectively (5). Published dried blood microsample-based methods for dasatinib had LLOQs of 2.5 ng/mL in 10-µL DBS (whole spot analysis) (41), 1 ng/mL in 40-µL DPS (43) and 0.5 ng/mL in 10-µL VAMS (35). Specificity is achieved by separation of the compounds on LC columns and detection in multiple reaction monitoring (MRM) mode. Several multi-TKI LC-MS/MS methods have been developed (35, 41, 43, 46), offering the advantage that one dedicated method can be applied for a variety of analytes (and, hence patient samples), which is especially relevant in routine clinical settings. Known disadvantages of LC-MS/MS are the high investment and maintenance cost, the high technical expertise that is required and the need for time-consuming sample preparation, which is typically more laborious for dried microsamples, when compared with plasma samples. However, LC-MS/MS analyses of dried blood microsamples can be fully automated using robotics, thereby allowing a higher throughput, decreasing the workload and improving turn-around time in the clinical laboratory (30). For example, the CAMAG DBS autosampler (Camag, Muttenz, Switzerland) allows 500 DBS cards to be stacked, which can subsequently be subjected to fully automated extraction and LC-MS/MS analysis: from card to result with no hands-on (52, 53). There are currently no published methods for TKI TDM using automated sample preparation. Automation of VAMS sample processing is also feasible, although at this point only rarely being employed, with no published reports available on routine implementation.

### c. Internal Standards

Stable isotopically labeled (SIL) internal standards (ISs) are regarded as the best choice to compensate for variations during sample preparation and LC-MS/MS analysis. The importance of using SIL-ISs was also observed in several dried blood microsample-based TKI methods: Antunes et al. found significant ion enhancement for imatinib in DBS, which was completely compensated using imatinib-D_8_ as IS (36). In our own work, we observed substantial ion suppression for most compounds measured in VAMS samples, which was also adequately compensated for by using SIL-ISs. Moreover, the ISs also compensated for losses during sampling preparation (35). A non-SIL IS was used in only one method: in the DBS method of Irie et al., erlotinib was used as IS for quantification of gefitinib, both being EGFR-TKIs with similar structures (40).

### d. Dilution of Dried Blood Microsamples

Patient samples with a TKI concentration above the measuring range should be diluted in order to obtain a correct quantitative result if clinically relevant. Whilst this is straightforward for plasma, this is less evident for dried microsamples, which cannot be directly diluted. Indeed, most published dried microsampling methods for TKI monitoring did not integrate dilution of samples as part of the validation. Nijenhuis et al. diluted DBS extracts with vemurafenib concentrations higher than the upper limit of quantification (ULOQ) (100 µg/mL) seven-fold with blank extracts (44). A similar approach was described for pazopanib: DBS extracts were diluted tenfold with a processed controlled matrix (45). Xu et al. described two different approaches to dilute DBS samples with MK-1775 concentrations above the assay ULOQ (1000 ng/mL). In the first method, blank DBS extract containing the IS was added to the DBS sample extract. The second approach involved mixing one subpunch from the DBS sample with five subpunches obtained from blank DBS. Both dilution methods provided accurate and reproducible results (27). If dilution of samples is to be performed in routine practice, dilution integrity should be an integral part of the method validation in order to report quantitative results above the highest point of the calibration line. Alternatively, clinically relevant ULOQs could be chosen for each TKI during method development. As a guidance, an ULOQ twice the upper limit of the therapeutic range can be considered adequate, with a reporting of “>ULOQ” when a signal above the ULOQ is encountered.

### e. External Quality Controls

When implementing TDM analyses in routine care, laboratories should participate in proficiency testing programs. To the best of our knowledge, there are currently no proficiency testing programs for TDM of TKIs in microsamples, while such a program readily exists for TDM of immunosuppressant drugs in microsamples, set up by the Dutch organization SKML (54). As an alternative, independent dried blood quality control (QC) samples can be generated using plasma-based (e.g. Asqualab) external QC samples (EQC) by replacing part of the plasma of a blank whole blood sample by the external QC material (31, 55, 56). The resulting blood sample can then be used to generate DBS. As the amount of plasma being replaced by EQC material is known, it is even possible to convert the obtained DBS concentration to a plasma concentration post-analysis, which can be formally reported to the proficiency test organizer.

### f. DBS Specific Validation Parameters

The use of DBS subpunches necessitates the evaluation of additional parameters during method validation. One aspect to be evaluated is whether the spotted volume of blood on the filter paper has an impact on the method accuracy. Nijenhuis et al. reported that for vemurafenib the volume of a blood drop affected the reproducibility of the method and volumes >20 µL increased the inaccuracy of the assay. However, the latter was considered clinically irrelevant as the authors stated that DBS samples collected *via* finger-prick typically correspond to blood volumes of approximately 10-20 µL (44). In other studies, for imatinib and norimatinib (39), pazopanib (45) or MK-1775 (27) no influence of spot sizes between 10 to 40 µL, 10 to 30 µL or 30 to 50 µL, respectively, were observed. Another aspect to be evaluated is the site of punching (central *versus* peripheral), as non-homogeneous distribution of drugs on filter paper has been described, commonly known as the “volcano effect” (48) (accumulation of red blood cells occurs at the very outer edge of a DBS). Several articles investigated this aspect by comparing results obtained from punches taken from the edge with those from punches taken at the center of a DBS. For imatinib and norimatinib (39), pazopanib (45) and MK-1775 (27) central and peripheral measurements gave equivalent results. Preferably, the volume and volcano effect should also be evaluated at different Hct and concentration levels (50).

### g. Stability

Theoretically, working with dried matrices improves the stability of most compounds. This allows a more cost-effective transport and storage: in many instances dried microsamples can be transported *via* regular mail (under ambient conditions) and stored for prolonged periods at room temperature (21). The advantage of improved stability was nicely exemplified for ibrutinib, which is only stable in plasma for a few hours, whereas in VAMS samples and DPS it was reported to be stable at room temperature for 2 (35) and 12 weeks (43), respectively. **Table 3** summarizes the reported stabilities of various TKIs in different types of microsamples. It should be noted that for TDM of TKIs long periods of sample storage are less relevant, as analyses typically need to be performed as quickly as possible, within 1 week (or less) after sampling. For TDM purposes it is mainly important that stability during the transport of the dried microsamples is guaranteed. Several authors investigated the stability of TKIs in dried microsamples stored at higher temperatures, to simulate extreme temperature conditions that could be encountered during transportation to the laboratory *via* regular mail (e.g. in a postbox fully exposed to the sun during summer): gefitinib was stable for 24h at 40°C (40), imatinib and nilotinib were stable for at least three days at 40°C (this was not the case for dasatinib) (41), imatinib for 36 days at 43°C (36) and most TKIs of two different TKI panels in VAMS for at least 2 days at 60°C (35, 46). It should be noted that not in all instances where instability was reported, the authors verified whether extractability wasn’t affected, hence resulting in *apparent* instability (57). For protection from humidity, dried microsamples are advised to be transported and stored in closed plastic bags with a desiccant. Drying also inactivates pathogens present in the blood, which reduces the biohazard risk (58).

**Table 3:** Reported stability of TKIs in different microsamples in alphabetical order of the first author.

Reference	TKI	Microsample	Storage condition
Antunes et al. (36)	Imatinib	DBS	36 days at -20, 25 and 43°C
Boons et al. (37)	Nilotinib	DBS	7 months in refrigerator (2-8°C)
de Wit et al. (38)	Pazopanib	DBS	75 days at ambient temperature
Iacuzzi et al. (39)	Imatinib	DBS	16 months at RT
	Norimatinib		
Irie et al. (40)	Gefitinib	DBS	5 months at RT
			5 months at -20°C
			24 hours at 40°C
Kralj et al. (41)	Imatinib	DBS	30 days at RT or -20°C
	Nilotinib		3 days at 40°C (except dasatinib)
	Dasatinib		
Mukai et al. (43)	Bosutinib	DPS	72 hours at 40°C/90% RH
	Dasatinib		12 weeks at RT
	Ibrutinib		
	Imatinib		
	Nilotinib		
	Ponatinib		
Nijenhuis et al. (26)	Vemurafenib	DBS	827 days at ambient temperature
Verheijen et al. (45)	Pazopanib	DBS	398 days at ambient temperature
Verougstraete & Stove (35)	Bosutinib	VAMS	1 month at -20°C, 4°C and RT (except ibrutinib 2 weeks at RT)
	Dasatinib		2 days at 60°C (except ibrutinib 1 day)
	Ibrutinib		
	Imatinib		
	Gilteritinib		
	Midostaurin		
	Nilotinib		
	Ponatinib		
Xu et al. (27)	MK-1775 (adavosertib)	DBS	6 months at -20°C
			14 months at ambient temperature
			8 days at 40°C/75% RH
Zimmermann et al. (46)	Afatinib	VAMS	6 weeks at RT (19% RH)
	Axitinib		2 days at 60°C (10% RH) (except afatinib and osimeritinib)
	Bosutinib		
	Cabozatinib		
	Dabrafenib		
	Lenvatinib		
	Nilotinib		
	Osimeritinib		
	Ruxolitinib		
	Trametinib		

DBS, dried blood spot; DPS, dried plasma spot; RH, relative humidity; RT, room temperature; TKI, tyrosine kinase inhibitor; VAMS, volumetric absorptive microsampling.

### h. Hematocrit Effects

For a variety of reasons, deviating Hct values may impact dried blood microsample-based quantitative results. The best-known effect of the Hct of a blood sample is related to the spreading of blood on filter paper, when working with conventional DBS collected *via* direct application of a drop of blood. This is related to the viscosity of the blood: blood with a higher Hct will spread less than blood with a lower Hct and will result in more concentrated spots. Hence, when a fixed-diameter punch is taken from DBS with a higher Hct, the volume of blood and thus the amount of analyte contained within this subpunch will be higher than in punches from DBS with a lower Hct. Spreading of the blood also depends on the type of filter paper used (47–49). For each DBS-based method, a Hct interval in which the impact of this ‘area bias’ is still acceptable should be established during method validation. This Hct effect is of particular importance in oncology patients, as the Hct may vary widely in this population: whereas the reported mean Hct of the different oncology study populations was quite constant, the ranges of these Hcts were wide (see **Table 2**). Furthermore, Hct values may also vary widely within the individual oncology patient due to concomitant chemotherapy, disease progression or overall clinical status.

Several approaches have been proposed that may allow to cope with the differential spreading of blood samples with varying Hct. A first approach implies the use of volumetrically applied DBS, followed by whole spot analysis. Kralj et al. followed this approach, after pipetting 10 µL EDTA whole blood onto filter paper (41). However, such accurate volumetric application of blood onto a filter paper is hard to envisage when aiming at home-sampling by the patients themselves. Another strategy is to determine or estimate the Hct of the blood used for DBS preparation, to correct for the Hct bias. For instance, Hct levels can be predicted by measuring the potassium concentration in DBS. Potassium concentrations correlate with the Hct because potassium is predominantly located intracellularly and erythrocytes are the prime cellular constituent of blood (59). Hct levels can also be predicted *via* determination of its total hemoglobin content, using non-contact single-wavelength reflectance spectroscopy (60, 61) or near infrared (NIR) spectroscopy (62–64). If the Hct level of the DBS (subpunch) is known, correction factors to alleviate the Hct bias can be used (61, 65) or plasma concentrations can be derived from the measured DBS concentrations (see further).

New devices, allowing an accurate volumetric collection of capillary dried blood microsamples, are continuously being developed. These maintain the benefits associated with DBS, while avoiding the above-described Hct-based area bias (66). One of the first strategies to achieve this is VAMS, which has evolved over the course of years into a valuable alternative to classical DBS sampling. VAMS devices (marketed as Mitra^®^ and introduced in 2014 by Neoteryx, Torrance, CA) consist of a porous absorbent polymeric tip, connected to a plastic handle. Upon touching a drop of blood for a few seconds, the tips absorb a fixed volume of blood (10, 20 or 30 µL) by capillary action, irrespective of the sample’s Hct (67, 68). Furthermore, compared to DBS, collection of VAMS samples has been scored as more simple and straightforward (69, 70). A current limitation of VAMS, when compared to DBS, is that there are hardly any reports on fully automated extraction and analysis – there is only one report describing the measurement of ten peptides in VAMS samples *via* an automated platform for protein extraction and digestion (71). As devices are being designed to enable easy, automated sample preparation by robotic liquid handling platforms (21), it is expected that VAMS samples will catch up with DBS in terms of automation for TDM analyses. For example, a Tecan^®^ (Männedorf, Switzerland) platform has already been used for automatic preparation of blood-dipped VAMS tips which can be used as calibrators or QCs. This only results in automation and time saving for the preparation of the VAMS samples themselves, with extraction and chromatographic analysis still requiring manual processes (72). To date, two groups have investigated the measurement of TKIs using VAMS, establishing multi-analyte methods for the simultaneous quantification of eight (35) or ten TKIs (46). Other volumetric microsampling devices avoiding the Hct and inhomogeneity bias include the HemaPEN (Trajan, Melbourne, Australia), Capitainer qDBS (Capitainer, Solna, Sweden) and HemaXis DB 10 (HemaXis, Gland, Switzerland) device (66). To the best of our knowledge, none of these devices has been used for the determination of TKIs yet. A drawback associated with these alternative sampling devices is the higher cost and, for some, the more complex sampling and processing technique compared to conventional DBS. These aspects, together with the recency of these developments, may explain the hitherto limited implementation of these devices in clinical routine (66).

Another approach to minimize the Hct bias effect is to prepare calibration standards in blood with a Hct level close to the expected Hct of the target population (30). For this approach the Hct among the target population and within the individual patients should be quite constant, which is not the case for the heterogeneous population of cancer patients treated with TKIs (see above). Moreover, one should know whether the Hct level of the sample lies within the covered Hct range – the above-mentioned Hct prediction strategies allow to derive this information. In the different published TKI methods the standards were prepared in blank blood with a Hct of 0.40 (35), 0.42 (41), 0.35 (36), 0.44 (45), 0.40 (37), 0.38 (39) or 0.45 (46).

In most of the published TKI microsample methods the influence of the Hct on the accuracy of the method has been evaluated by preparing QC samples at different Hct levels and comparing the obtained results to those obtained from QC samples with the Hct value of the calibrators. No significant impact for the following Hct ranges on the accuracy was observed for imatinib [0.25-0.50] (36), imatinib combined with its active metabolite norimatinib [0.29-0.59] (39), imatinib included in a multi-TKI method together with dasatinib and nilotinib [0.30-0.60] (41), MK-1775 [0.16-0.85] (27), nilotinib [0.25-0.50] (37), pazopanib [0.20-0.65] (38) and [0.35-0.50] (45), vemurafenib [0.24-0.45] (44) and for a multi-analyte method containing eight [0.18-0.55] (35) and ten [0.30-0.55] (46) TKIs.

The second effect of the Hct on dried blood microsampling is its potential impact on the recovery of the analyte from the dried blood microsample. This is the case for both conventional DBS and for the newer microsampling devices: Hct-dependent recoveries have been described for DBS (73) and can even be more profound for VAMS samples (74, 75). Most often, high Hct levels have a negative effect on the analyte recovery, resulting in an underestimation of the analyte concentration (76). The latter has been explained for VAMS samples by the larger amounts of erythrocytes present at higher Hct levels, which may trap the analytes in the pores of the VAMS tip, resulting in a lower recovery (75). Similarly, the recovery may be impacted upon ageing of the dried samples. Therefore, thorough optimization of the extraction procedure during method development is critical to ensure the robustness of a dried blood microsample-based method. Besides the use of different extraction solvents and elevated temperatures to obtain a more robust Hct-independent extraction (55, 56), Mano et al. concluded that the inclusion of a sonication step helps to improve the extraction of the compounds from the porous VAMS material (77). Importantly, as IS are typically added together with the extraction solvent, these will only compensate for post-extraction biases and will not compensate for recovery issues. The latter can potentially be solved for DBS analyses by spraying the IS onto the filter paper before sample extraction (78). However, in all the published TKI DBS methods, the ISs were added to the extraction solvent or directly added to the sample before extraction (12, 27, 36–41, 43–45). In a multi-analyte TKI method on VAMS samples clear Hct-dependent recovery issues were observed over a Hct range from 0.20 to 0.60 at different storage conditions (i.e. -80°C, room temperature and 60°C) during pre-validation stress testing. The issues were eventually resolved by developing a rigorous extraction protocol including pre-wetting of the VAMS tips, sonication and liquid-liquid extraction followed by an evaporation step. The robustness to the impact of Hct was formally demonstrated during method validation, where IS-compensated recoveries from VAMS samples, derived from blood with Hct ranging from 0.18 to 0.55, ranged from 83 to 125%, with all CVs being less than 11.6% (35). Next to its effect on recovery, samples with different Hct levels can also be considered as different matrices, with the potential to give rise to Hct-dependent matrix effects (MEs). It is therefore recommended that during method validation, next to the blank matrices obtained from six different individuals, blood samples covering a broad Hct range should also be included for the evaluation of MEs (50). When evaluating MEs and recovery of the assay, it is important that these are consistent, rather than being minimal (for ME) or maximal (for recovery). We demonstrated in our published TKI VAMS method that MEs and recoveries were Hct-independent over a broad Hct range (0.18-0.55) (35).

Lastly, the Hct of a sample may also influence the blood-to-plasma concentration ratio and thus the blood to plasma concentration conversion (79). The different approaches for converting an obtained dried blood result to a plasma concentration will be discussed thoroughly in the following section.

### i. (Dried) Blood to Plasma Conversion

TKI target ranges are typically plasma-based, implying that for clinical interpretation dried blood matrix results should be converted to plasma concentrations. During (clinical) method validation a correlation study between DBS (blood) and plasma concentrations should be established using authentic patient samples. Most publications comparing TKI concentrations derived from DBS with those from corresponding plasma reported deviating (but correlating) concentrations. For those TKIs, the DBS concentrations should be converted to plasma concentrations. Equivalent concentrations were reported for gefitinib only for finger-prick DBS and venous plasma measurements, so for that analyte plasma results can be directly replaced by DBS concentrations without applying a conversion method (40). This means that gefitinib is probably slightly more partitioned in blood cells than in plasma, as upon equal partitioning a blood concentration is typically slightly lower than a plasma concentration.

In literature, dried blood TKI concentrations were converted to plasma concentrations based upon approaches that take into account a Hct correction, with or without considering the influence of blood-to-plasma partitioning of the TKI, or directly *via* empirically obtained regression equations or correction factors. When applying such experimentally determined correction factor or regression, it is essential that this is validated on an independent dataset which was not used for deriving that factor (80). Nilotinib plasma concentrations derived from capillary DBS concentrations were calculated, taking into account the Hct and the blood-to-plasma ratio using the formula described by Wilhelm et al. (30). Additionally, the plasma nilotinib concentrations were predicted based on an empirically obtained Deming regression equation. Both methods showed similar predictive performance and both could be used to predict plasma concentrations from DBS nilotinib concentrations (37). Vemurafenib plasma concentrations were predicted *via* a comparable Hct-based conversion method, taking into account the blood cell to plasma partition coefficient. The plasma concentrations were also appropriately predicted *via* an experimentally obtained regression equation (26). Alternatively, Antunes et al. took into account the fraction of the drug in plasma (f_p_) for estimating imatinib plasma concentrations (C_p_): C_p_= [C_DBS_/(1-Hct)] x f_p_. The f_p_ imatinib value was determined as the value which resulted in a mean ratio between measured and estimated imatinib plasma concentration of 1. In line with the other studies, this group also set up a conversion using a correction factor to directly convert DBS concentrations to plasma concentrations without considering the Hct value nor other variables: both conversion methods resulted in a high agreement between calculated and measured imatinib plasma concentrations (36). Pazopanib (38), imatinib and norimatinib (39) and imatinib, nilotinib and dasatinib (41) plasma concentrations were calculated from DBS concentrations *via* a Hct correction using the formula: C_p_= C_DBS_/(1-Hct), which neglects the possible distribution of TKIs into the blood cells. This formula can be used for TKIs which are only present in the plasma compartment or for TKIs with a high protein binding (for example pazopanib >99.9%), since only the unbound fraction of TKIs can partition into blood cells and the unbound fraction can be considered to be negligible (38). Iacuzzi et al. compared the simple Hct-conversion method with a conversion *via* an experimentally determined correction factor and found that the latter yielded a slightly better agreement with the measured plasma concentrations (39). Verheijen et al. applied an empirical Deming regression equation to convert pazopanib DBS to plasma concentrations. Correction for individual Hct did not improve the correlation between calculated and measured plasma concentrations (45). For radotinib, an approach was proposed in which plasma concentrations were calculated by using the measured Hct value in a second-degree polynomial function (42). When a Hct-based correction method is used, specific measured or predicted Hct levels or a fixed Hct applicable for the whole intended study population can be used (37, 38). The latter approach has the advantage that the individual Hct level of a microsample should not be measured or estimated, however this requires a population with rather homogenous Hct values. We previously determined the mean ratio between actual plasma and whole blood concentrations for TKIs used for treatment of hematological malignancies (i.e. bosutinib, dasatinib, gilteritinib, ibrutinib, imatinib, midostaurin, nilotinib and ponatinib) and found substantial differences, both for different TKIs, as well as (for certain TKIs) between different individuals, although larger patient sets are required to substantiate these findings (23). Similarly, Zimmermann and colleagues determined VAMS whole blood to plasma conversion factors for a panel of ten TKIs (i.e. afatinib, axitinib, bosutinib, cabozantinib, dabrafenib, lenvatinib, nilotinib, osimertinib, ruxolitinib and trametinib), noting that for cabozantinib, dabrafenib, lenvatinib, nilotinib, osimertinib and ruxolitinib the Hct impacted the VAMS whole blood to plasma concentration conversion. However, it should be noted that the latter experiments were performed *in vitro* on spiked blood samples and not on authentic patient samples (46). The obtained blood-plasma ratios are still to be evaluated clinically in elaborate dried blood microsample *versus* plasma comparison studies.

Using plasma instead of whole blood for the generation of dried matrix spots, resulting in DPS, allows to overcome the Hct effects and, in addition, overcomes the need to calculate a plasma concentration from a (dried) blood concentration. The availability of DPS should allow smooth application, without any need for conversion. Mukai et al. developed and validated an analytical method for the simultaneous quantification of bosutinib, dasatinib, ibrutinib, imatinib, nilotinib and ponatinib in DPS. DPS were generated by pipetting 20 µL of plasma onto a filter paper. Despite expecting to be equivalent matrices, significant systematic errors were observed between plasma and DPS concentrations for bosutinib, nilotinib and ponatinib, suggesting a methodological issue (43). Since the plasma was prepared by centrifugation of whole blood, this approach is not feasible for home-sampling. It only retains the benefits of more convenient sample transport and storage. Plasma separator devices which can generate volumetric DPS from a non-volumetrically applied drop of blood onto multilayered filter membranes are currently commercially available. While these devices have the advantage that the need for a centrifugation step is eliminated, only few applications have been published (81, 82). A disadvantage of DPS, compared to dried blood microsamples, is that for the generation of DPS higher sample volumes are required.

Last, for several TKIs with a high protein binding, the monitoring of the free TKI fractions can be theoretically more relevant as only this fraction is likely to penetrate into the tumor cells and induce pharmacologic effects. The high protein bound TKIs are predominantly bound to albumin and α-acid glycoprotein, which means that fluctuating levels of these proteins influence total TKI concentrations (6, 18). This poses an additional limitation for dried blood microsamples as -per definition- these can only be used for measuring total TKI concentrations. However, there is to date still a lack of consensus regarding the utility of unbound TKI measurements.

## Towards Home-Sampling

Given the simple sample collection and stabilizing effect on the analyte, microsamples can be collected at home and can be sent to the laboratory *via* regular postal services under ambient conditions, prior to consultation. This allows the actual TKI level of the patient to be available at the time of consultation, allowing insight into the most recent data. As described earlier, TKI targets are generally based on trough concentrations. Obtaining trough levels is definitely not always easy when a patient has to come to the hospital or needs to visit a doctor for a blood draw. Home-based sampling is superior in this respect: within the comfort of the home-setting, the patient can self-collect a sample at the right time point. This will also prevent the risk of delay in drug intake when the patient should be sampled at a medical center. However, home-sampling is only feasible if the patients are clearly instructed and received adequate training about sample collection and, importantly, are aware of the importance of a correct sample collection (69, 70, 83, 84).

Most TKI studies artificially prepared dried microsamples in the lab by pipetting venous blood on filter paper (27, 35, 41, 43) or had DBS samples collected by trained personnel in a hospital environment (26, 28, 36–39, 42, 44, 45). In the study of Irie et al. the skin punctures were performed by the patients themselves, although it was not specified whether this was supervised or not and in which environment (40). Based on a questionnaire taken by Antunes and colleagues, patients preferred DBS collection over venous sampling as sampling method for performing TDM of imatinib (36). Boons et al. examined the feasibility of nilotinib DBS self-sampling for CML patients in a real home-sampling context. They concluded that DBS self-sampling is feasible in clinical practice after giving adequate sampling instructions. Moreover, the patients believed in the reliability of DBS self-sampling and considered the sampling easy and not painful. It should be noted that these authors only reported on the feasibility of applying such a sampling strategy in a home-sampling context, actual results from nilotinib concentrations or a correlation with clinical parameters were not reported (83). In a very recent study performed by Zimmermann et al., capillary VAMS samples were successfully collected by either healthcare professionals or patients at home for the determination of afatinib, cabozantinib, dabrafenib, trametinib, nilotinib and ruxolitinib, readily providing a first indication of real-life applicability (46).

Despite the above-mentioned blood *versus* plasma interpretation issues, dried blood microsamples are typically taken by finger-prick in the home-environment. This means that capillary blood is used instead of venous blood, which may give rise to different concentrations (58). Differences between capillary and venous blood concentrations can be expected shortly after administration, during the (absorption and) distribution phase of the drug (79). Several publications report a good agreement between concentrations from finger-prick DBS samples and DBS samples generated by pipetting venous blood on the card, as exemplified for imatinib (and norimatinib) (39), pazopanib (38) and R406 (metabolite fostamatinib) (28). This suggests equivalence between capillary and venous blood concentrations for these components. However, for most TKIs such equivalence still needs to be examined.

We are not aware of any method using dried blood microsamples for TKI quantitation that has been implemented in routine clinical practice. An important factor that may hamper such implementation is the additional cost (for the lab) that may be associated with analyzing dried blood microsamples instead of conventional liquid blood or plasma samples. Examples of costs associated with dried blood microsample analysis include: a more extensive method development and validation, the set-up of comprehensive sample preparation protocols, shipping costs for the samples, as well as the cost of the microsampling device itself and lancets to perform a finger-prick. In this context, an intrinsic hurdle is often posed by the fact that the analyzing lab itself may not profit from the advantages (e.g. home-based sampling overcoming the need to come to a hospital) and cost savings (e.g. no specialized staff required for sampling) associated with dried blood microsampling, while being responsible for providing collection devices (with microsampling devices being more expensive than regular blood tubes). Furthermore, despite the above-mentioned methodological aspects inherent to dried blood microsamples, some other factors could be optimized to allow smoother implementation in clinical routine. E.g., the availability of fully automated methods/analyzers that are compatible with the workflow of clinical labs could help to decrease hands-on time and increase throughput of dried blood microsamples. Another example is the set-up of EQC programs enabling accreditation of lab-developed methods in clinical labs (85).

## Conclusions/Future Perspectives

Despite the growing evidence of the relevance for performing TDM of TKIs in oncology patients, it is still not integrated in the standards of care. Microsampling can be a useful tool to promote and further elaborate on studies of TDM of TKIs. Several papers have already been published in which TKI microsample-based methods are described, with most of these focusing on DBS. Despite the many benefits that are associated with the use of dried blood microsamples, a few issues should be tackled during method development and validation. Therefore, thorough optimization and evaluation of microsample-based analytical methods is essential. In the future, studies should be set up to confirm exposure-response relationships and to define therapeutic ranges for all TKIs. Subsequently, the use of TDM of TKIs in clinical oncology practice must be evaluated. For implementation of TDM in a home-sampling context, real-life home-sampling studies must be set up to evaluate the feasibility in specific oncology patient groups. To date there is no dried blood microsample-based TKI method used for TDM in routine yet. While we are on the right track to implement TDM of TKIs in standard care of oncology patients, we are not there yet, and using dried blood microsamples can be a useful tool to help to reach this goal.

## Author Contributions

NV wrote the manuscript. VS edited the manuscript. AV edited the manuscript. CS, supervisor, wrote the manuscript. All authors contributed to the article and approved the submitted version.

## Funding

This work was supported by the Research Foundation-Flanders (FWO) (grant number 1703320N) to NV. 

## Conflict of Interest

The authors declare that the research was conducted in the absence of any commercial or financial relationships that could be construed as a potential conflict of interest.

## Publisher’s Note

All claims expressed in this article are solely those of the authors and do not necessarily represent those of their affiliated organizations, or those of the publisher, the editors and the reviewers. Any product that may be evaluated in this article, or claim that may be made by its manufacturer, is not guaranteed or endorsed by the publisher.

## Abbreviations

AML, acute myeloid leukemia; CML, chronic myeloid leukemia; DBS, dried blood spot; DPS, dried plasma spot; EDTA, ethylenediaminetetraaectic acid; EQC, external quality control; GIST, gastrointestinal stromal tumor; Hct, hematocrit; IATDMCT, International Association of Therapeutic Drug Monitoring and Clinical Toxicology; IS, internal standard; LC-MS/MS, liquid chromatography tandem mass spectrometry; LLOQ, lower limit of quantitation; ME, matrix effect; MRM, multiple reaction monitoring; NIR, near infrared; NSCLC, non-small-cell lung carcinoma; QC, quality control; RCC, renal cell carcinoma; SIL, stable isotopically labeled; SKML, stichting kwaliteitsbewaking medische laboratoriumdiagnostiek; TDM, therapeutic drug monitoring; TKI, tyrosine kinase inhibitor; ULOQ, upper limit of quantification; VAMS, volumetric absorptive microsampling.
